# Pathologic Replication-Independent Endogenous DNA Double-Strand Breaks Repair Defect in Chronological Aging Yeast

**DOI:** 10.3389/fgene.2018.00501

**Published:** 2018-10-25

**Authors:** Monnat Pongpanich, Maturada Patchsung, Apiwat Mutirangura

**Affiliations:** ^1^Department of Mathematics and Computer Science, Faculty of Science, Chulalongkorn University, Bangkok, Thailand; ^2^Center for Excellence in Molecular Genetics of Cancer and Human Diseases, Chulalongkorn University, Bangkok, Thailand; ^3^Department of Anatomy, Faculty of Medicine, Chulalongkorn University, Bangkok, Thailand

**Keywords:** replication-independent endogenous DNA double-strand breaks, repair defect, modified ends with insertion, *Saccharomyces cerevisiae*, chronological aging

## Abstract

Reduction of physiologic replication-independent endogenous DNA double strand breaks (Phy-RIND-EDSBs) in chronological aging yeast increases pathologic RIND-EDSBs (Path-RIND-EDSBs). Path-RIND-EDSBs can occur spontaneously in non-dividing cells without any inductive agents, and they must be repaired immediately otherwise their accumulation can lead to senescence. If yeasts have DSB repair defect, retention of Path-RIND-EDSBs can be found. Previously, we found that Path-RIND-EDSBs are not only produced but also retained in chronological aging yeast. Here, we evaluated if chronological aging yeasts have a DSB repair defect. We found a significant accumulation of Path-RIND-EDSBs around the same level in aging cells and caffeine treated cells and at a much higher level in the DSB repair mutant cells. Especially in the mutant, some unknown sequence was found inserted at the breaks. In addition, % difference of cell viability between HO induced and non-induced cells was significantly greater in aging cells. Our results suggested that RIND-EDSBs repair efficiency declines, but is not absent, in chronological aging yeast which might promote senescence phenotype. When a repair protein is deficient, an alternative pathway might be employed or an end modification process might occur as inserted sequences at the breaks were observed. Restoring repair defects might slow down the deterioration of cells from chronological aging.

## Introduction

We previously proved that pathologic endogenous DNA double-strand breaks (EDSBs) can occur spontaneously, even without exposure to radiation or DNA damaging agents, and were excessively detectable when non-dividing cells had functional double-strand breaks (DSB) repair defects. In other words, they are produced independently of replication, hence we named them pathologic replication-independent EDSBs (Path-RIND-EDSBs) (Thongsroy et al., 2018). They are lethal and must be repaired just as other DNA lesions (Hoeijmakers, 2009). They are associated with a decrease in the cell’s viability (Thongsroy et al., 2013). In addition, the findings that γ-H2AX foci were observed in mice brains supported the existence of Path-RIND-EDSBs in non-dividing cells (Barral et al., 2014; Hare et al., 2018). When non-dividing cells age, they are produced in large numbers, possibly due to a reduction of Physiologic RIND-EDSBs (Phy-RIND-EDSBs) another type of RIND-EDSBs. Phy-RIND-EDSBs, in contrast to Path-RIND-EDSBs, were shown to possess a functional role as epigenetic markers as they affected genome integrity and were generated by non-random mechanism (Pornthanakasem et al., 2008; Thongsroy et al., 2018). Previous studies showed evidence that a reduction of Phy-RIND-EDSBs decreased cell viability and promoted genome instability (Thongsroy et al., 2018). In addition, they are preferentially located in hypermethylated genome regions, were retained in facultative heterochromatin in human cells, preferentially occurred immediately after certain 4-bp sequence and were prevented from occurring after certain 4-bp in yeast (Kongruttanachok et al., 2010; Pongpanich et al., 2014).

RIND-EDSBs can be divided into three groups: odds ratio (OR) > 1 breaks, OR ≤ 1 breaks and modified ends with insertion at the breaks (MIB) (Thongsroy et al., 2018). OR > 1 breaks are breaks that were found more frequently in wild type; therefore, they represent Phy-RIND-EDSBs. OR ≤ 1 breaks are breaks that were unlikely to be found in wild type. MIB are breaks that had a certain sequence inserted. In yeast, JKM179 treated with caffeine (a repair inhibitor), OR ≤ 1 breaks and MIB increased (Thongsroy et al., 2018). Therefore, OR ≤ 1 breaks and MIB represent Path-RIND-EDSBs.

In our previous work, we found that in a condition where cells possessed a low level of Phy-RIND-EDSBs and were treated with chemical to inhibit repair (caffeine), Path-RIND-EDSBs and viability reduction was significantly greater than cells with only low level of Phy-RIND-EDSBs (Thongsroy et al., 2018). We have shown that aging cells have a low level of Phy-RIND-EDSBs which cause a high level of Path-RIND-EDSBs. Therefore, if aging cells are decreased in DSB repair function, production and retention of Path-RIND-EDSBs can promote DNA damage response leading to cellular senescence.

The general notion has been that DNA repair machinery is becoming more compromised and error-prone with age (Gorbunova et al., 2007; Li and Vijg, 2012). Major DNA repair pathways are base excision repair (BER), nucleotide excision repair (NER), mismatch repair (MMR), and double-strand break repair (DSBR). The repair pathway used depends on the type of lesion (Maynard et al., 2015). BER repairs DNA lesions such as oxidation products generated by ROS; NER removes bulky lesions; MMR removes mismatches in DNA and DSBR works on double-strand break (Gredilla, 2011). Numerous studies have found a significant decline of all four major repair pathways activities: BER (Intano et al., 2002, 2003; Zhang et al., 2010; Løhr et al., 2015; Xu et al., 2015), NER (Goukassian et al., 2000; Boyle et al., 2005; Yamada et al., 2006), MMR (Yehuda et al., 2001; Neri et al., 2005) and DSBR (Seluanov et al., 2004; Ju et al., 2006; Vyjayanti and Rao, 2006; Mao et al., 2012; Vaidya et al., 2014; Li et al., 2016; Delabaere et al., 2017) with aging. Increasing evidence suggests that deficiencies in BER, NER, and DSBR indeed induce aging-associated phenotypes (Borgesius et al., 2011; Pan et al., 2016; Guedj et al., 2017). Werner syndrome patients with an inherited defect in BER show features of premature aging (Pan et al., 2016). Reduction of the activity of enzymes involved in mitochondrial BER has been shown to promote aging (Pan et al., 2016). Defects in NER cause three distinct premature aging disorders in human: xeroderma pigmentosum, Cockayne’s syndrome and trichothiodystrophy (Coppede and Migliore, 2010). Trichothiodystrophy (TTD) mice, defective in NER, exhibit phenotypes associated with advanced aging, including osteoporosis, osteosclerosis, gray hair, cachexia, and reduced life-span (de Boer et al., 2002). *Xpd*^m/m^ mice, which have a partial defect in both GG-NER and TC-NER, exhibited prominent premature aging features and about 20% reduction in lifespan (Dollé et al., 2006). Mice with a defect in Ku80 (Ku86), a NHEJ gene, exhibit an early onset of aging characteristics (Hasty et al., 2003).

Many studies have found a significant decline of double-strand break repair (DSBR) activities with aging (Seluanov et al., 2004; Ju et al., 2006; Vyjayanti and Rao, 2006; Mao et al., 2012; Vaidya et al., 2014; Li et al., 2016; Delabaere et al., 2017). Two major mechanisms of DSBR are homologous recombination (HR) and non-homologous end joining (NHEJ). HR repair has been shown to decline with increasing replicative age and can be restored by supplementation with SIRT6 (Mao et al., 2012). Rad51 does not change in expression level with age (Li et al., 2016); however, age-related HR defects are possibly caused by malfunctions of steps after Rad51 recruitment (Delabaere et al., 2017). NHEJ efficiency declines with age (Seluanov et al., 2004). End joining activity became less efficient (Vyjayanti and Rao, 2006) and its fidelity declined with age as it was associated with extended deletions and insertions in old cells (Seluanov et al., 2004; Vaidya et al., 2014). In addition, the frequency of microhomology-mediated end joining (MMEJ) increased with age, suggesting that as NHEJ declined in function it was compensated by MMEJ (Vaidya et al., 2014). It has been shown that declining DSB repair is associated with decreased expression of Ku70 and Mre11 (Ju et al., 2006). Another study showed that restoration of XRCC4 and Lig4 significantly promotes the NHEJ fidelity and efficiency in aged fibroblasts (Li et al., 2016). Although there are many studies on aged-related changes in DSB repair, DSB repair defect in chronological aging cell has not been extensively studied.

In this work, we aim to find evidence that DSB repair defect occurs in chronological aging yeast and in turn causes retention of Path-RIND-EDSBs. To study age-related changes in RIND-EDSBs repair, we perform sequencing at RIND-EDSBs in cells treated with caffeine, repair deficient mutant yeast and chronologically aging yeast.

## Materials and Methods

### Yeast Strain, Media and Growth Condition

Yeast strains used in this study were BY4741 (*MATa his3Δ1 leu2Δ0 met15Δ0 ura3Δ0*) as wild type, *mec1Δ* (*MATa his3Δ1 leu2Δ0 met15Δ0 ura3Δ0 mec1Δ sml1Δ*), *mre11Δ* (*MATa his3Δ1 leu2Δ0 met15Δ0 ura3Δ0 mre11Δ::KanMX*), *nej1Δ* (*MATa his3Δ1 leu2Δ0 met15Δ0 ura3Δ0 nej1Δ::KanMX*), *rad51Δ* (*MATa his3Δ1 leu2Δ0 met15Δ0 ura3Δ0 rad51Δ::KanMX*), *tel1Δ* (*MATa his3Δ1 leu2Δ0 met15Δ0 ura3Δ0 tel1Δ::KanMX*), *yku70Δ* (*MATa his3Δ1 leu2Δ0 met15Δ0 ura3Δ0 yku70Δ::KanMX*), *yku80Δ* (*MATa his3Δ1 leu2Δ0 met15Δ0 ura3Δ0 yku80Δ::KanMX*), *nhp6aΔ* (*MATa his3Δ1 leu2Δ0 met15Δ0 ura3Δ0 nh6aΔ::KanMX*) and *JKM179* (*MATα Δho hmlΔ::ADE1 hmrΔ::ADE1 lys5 leu2 ura3 trp1ade3::GALHO*) from the same sources as previously described (Thongsroy et al., 2013; Pongpanich et al., 2014). Asynchronous yeast cultures were grown in YPD (Sigma, United States) until an optical density (OD) at 600 nm reached 0.4–0.6 (cell density ∼0.53–0.87 × 10^7^ cells/ml) then yeast cells were arrested at G0 phase by culturing in YP medium containing 2% raffinose (Sigma, United States) for 2 days. Cells in the G0 phase that were small and without buds were confirmed by their morphology under a microscope. All cells of all yeast strains consisted of G0 phase cells (100% unbudded stationary-phase cells). Cell viability in all experiments was monitored by following these steps. First, cell density of yeast cultures was determined by measuring OD600. This value was used to calculate the volume required for making 1 ml of 10^6^ cells/ml dilute solution. Then ten-fold serial dilutions was performed to produce 1 ml of 10^3^ cells/ml solution. An aliquot of 100 μl of 10^3^ cells/ml solution (approximately 100 cells) was plated on a YPD plate and counting the number of visible colony forming units (CFUs). To inhibit the ATM/ATR-dependent DNA repair pathways, yeast cells were treated with 10 mM caffeine (Sigma, United States) for 24 h (Moser et al., 2000).

For the two chronological aging experiments (experiment 1: wild type and wild type day 50; experiment 2: wild type, *yku70Δ, nej1Δ and nhp6aΔ*), yeast cells were grown in YP + 2% glucose at 30°C 250 rpm overnight and then switched to YP + 2% raffinose for 2 days before being washed and resuspended in sterile deionized water. The cell pellets were collect at the first day in water (day 1) for all strains, the next 10 days (day 10) for wild type, *yku70Δ, nej1Δ and nhp6aΔ* and the next 50 days (day 50) for wild type day 50. % cell viability was calculated using the following formula (Figure 3)

$$\%\;\mathrm{cell}\;\mathrm{viability}=\frac{\mathrm{number}\;\mathrm{of}\;\mathrm{cells}\;\mathrm{at}\;\mathrm{day}\;10}{\mathrm{number}\;\mathrm{of}\;\mathrm{cells}\;\mathrm{at}\;\mathrm{day}\;1}\times 100$$

**FIGURE 1:**
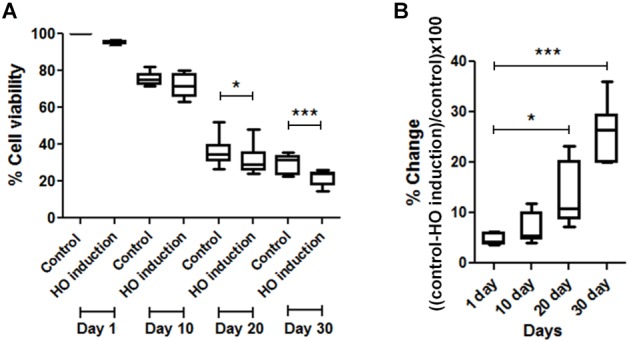
Comparisons of cell viability between induced and non-induced cells every 10 days. **(A)** Percent viability in JKM179 with and without induction every 10 days. There was a significant difference of % cell viability between control and induced cells at day 20 (^∗^*P* < 0.05) and day 30 (^∗∗∗^*P* < 0.001) **(B)** % difference of cell viability between JKM179 with and without induction HO induction every 10 days. There was a significant increase between day 1 and day 20 (^∗^*P* < 0.05) and day 1 and day 30 (^∗∗∗^*P* < 0.001) of % difference of cell viability between induced and non-induced cells.

For the HO induction experiment, JKM179 was used (Kim et al., 2007). Expression of the HO endonuclease was induced by the addition of galactose to the media for 3 h to produce a DSB in the yeast genome. After induction, cells were placed in media containing 2% glucose to repress HO expression and washed with sterile deionized water. Approximately 1 × 10^8^ cells/ml were resuspended in sterile deionized water (0 hr) and maintained at 30°C in a rotary shaker at 250 rpm until the end of the experiment. After HO induction, cell viability was measured by plating an aliquot of the culture on a YPD plate and counting the number of visible colony forming units (CFUs). Cell viability was monitored every 10 days until 30 days. % cell viability was calculated using the following formula (Figure 1A)

$$\%\;\mathrm{cell}\;\mathrm{viability}=\frac{\mathrm{number}\;\mathrm{of}\;\mathrm{HO}\;\mathrm{induced}\;\mathrm{cells}}{\mathrm{number}\;\mathrm{of}\;\mathrm{control}\;\mathrm{cells}}\times 100$$

% difference of cell viability between induced and non-induced cells (control) was calculated from (Figure 1B)

$$\%\;\mathrm{difference}\;\mathrm{of}\;\mathrm{cell}\;\mathrm{viability}=\frac{(\%\;\mathrm{viability}\;\mathrm{of}\;\mathrm{control}-\%\;\mathrm{viability}\;\mathrm{of}\;\mathrm{HO}\;\mathrm{induction)}}{\%\;\mathrm{viability}\;\mathrm{of}\;\mathrm{control}}\times 100$$

### High-Molecular Weight (HMW) DNA Preparation

High Molecular weight DNA preparation was performed as previously described (Pongpanich et al., 2014). In brief, yeast cells were treated with 1 mg/ml lyticase for yeast cell wall degradation, mixed with 1% low melting point agarose and embedded in plug molds. The yeast cell plugs were digested in a cell membrane digesting buffer, treated with T4 DNA polymerase to polish the cohesive end EDSBs. Then we performed the first linker ligation, digested the first linker ligated DNA with *RsaI* and ligated again with the second linkers.

### Library Preparation and Sequencing

Library preparation and EDSB sequencing were performed as previously described (Pongpanich et al., 2014). In brief, to detect RIND-EDSBs, the first linker was ligated to one of the EDSBs end. Note that, because the size of the first linker-ligated DNA was long, we added *RsaI*, which does not cut within the linker, to digest the first linker-ligated DNA. After digestion, the second linker is ligated. Therefore, the intact sequence would contain the first linker, the DNA sequence and the second linker. HMW DNA with two-linker ligation was then subjected to PCR and sequenced on an Ion Torrent sequencer (Ion Torrent^TM^ Personal Genome Machine^®^ (PGM), Life Technologies, United States).

### Data Pre-processing and Mapping

There are two linkers: the first is ligated to EDSBs and the second is ligated to *RsaI* digested DNA. The first linker is composed of two parts: 5′ end have the same sequence as primer for amplification and 3′ end is a unique sequence. For each sample, we extracted the sequence following the intact 3′ end of first linker sequence by first aligned reads and their reverse complement against the first linker sequence to determine the strand. The aligned position was used to determine the position that sequence was extracted. As there was a heterogeneity in the PCR product, we retained only reads with intact 3′ end of the first linker sequence. Only reads with intact 3′ end of first linker sequence contained RIND-EDSBs, which occur between the last base of linker sequence and its next base. Then we aligned the retained reads against the *Saccharomyces cerevisiae* strain B4741 reference genome, downloaded from the Saccharomyces Genome Database (SGD) using Nucleotide-Nucleotide BLAST 2.3.0+ (Altschul et al., 1990). Thus the first base of aligned reads corresponded to the position after breaks.

Using the BLAST results, we divided reads into two groups based on the position of the first base that got mapped. The first group contained reads that got mapped from the first base and the second group contained reads that were not mapped from the first base. For the second group, we interpret the unaligned portion as an inserted sequence at the break and called them modified ends with insertion at the breaks (MIB). The unmapped portion is extracted and shown in Supplementary Tables 1–10. Regardless of the mapped position of the first base, reads could be uniquely mapped or mapped to multiple locations (multi-mapped reads). We retained multi-mapped reads if the prior four bases of 90% of the mapped location were all the same.

### Data Analysis

#### OR > 1 Breaks, OR ≤ 1 Breaks and MIB Calculation

We counted the number of reads that could not be mapped from the first base and quantified them as MIB. Then we extracted the 4-bp sequence immediately prior to the breaks for each read, whether those reads were mapped from the first base or not. Then we counted the reads by prior 4-bp for all possible combinations of 4-bp (i.e., AAAA, AAAC and so on, denoted by XXXX) for reads mapped from the first base and did the same thing for reads not mapped from first base in all samples.

Next, we counted the occurrence of each possible combination of 4-bp sequence in the reference genome. Two hundred and fifty-six 2 × 2 contingency tables were then constructed for wild type only, one table per one 4-bp sequence. For each table the first column represents the specific 4-bp sequence; the second column represents all other possible 4-bp sequences. The first row represents the number of reads mapped from first base in our sample; the second row the number of occurrence in the reference genome. The tables were formulated as below.

**Table:** 

A: the number of reads mapped from first base that had the specific XXXX sequence	B: the number of reads mapped from first base that had the rest of the 4-bp sequences
C: the number of occurrence of the specific XXXX sequence in the reference genome	D: the number of occurrence of the rest of the 4-bp sequences in the reference genome


where XXXX represents each possible combination of 4-bp (i.e., AAAA, AAAC and so on) resulting in 256 tables in total.

OR was computed from these contingency tables using

$$Odds\;ratio=\frac{A\times D}{C\times B}$$

**FIGURE 2:**
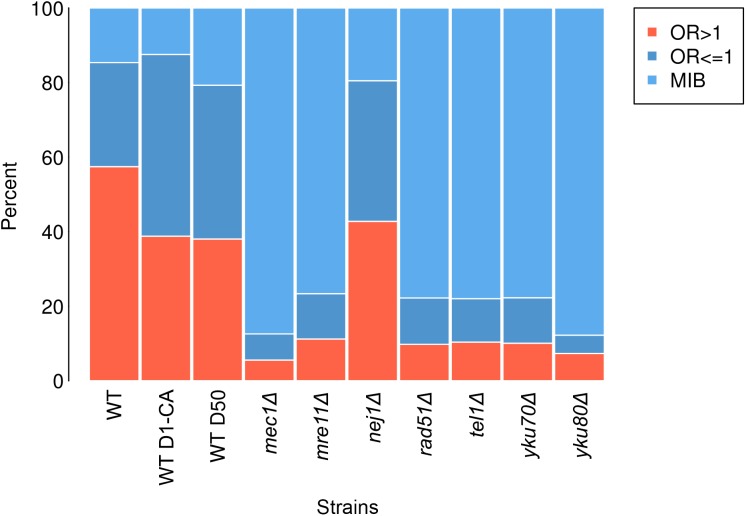
Percentage of each break type—OR > 1 breaks (salmon), OR ≤ 1 breaks (dark blue) and MIB (light blue) in wild type (WT), wild type treated with caffeine (WT D1-CA), wild type day 50 (WT D50), *mec1Δ, mre11Δ, nej1Δ, rad51Δ, tel1Δ, yku70Δ*, and *yku80Δ*.

Any break in wild type which had prior 4-bp sequence with OR > 1 was regarded as an OR > 1 break and any 4-bp sequence with OR ≤ 1 was regarded as an OR ≤ 1 break. A percentage of reads that had the same prior 4-bp sequences as sequences in OR > 1 breaks and OR ≤ 1 in wild type was calculated in all samples (Figure 2).

#### Statistical Analyses

To determine whether having repair defect increased the number of OR ≤ 1 breaks and MIB significantly, we performed Fisher’s exact tests (Table 1). We calculated nine 2 × 2 contingency tables considering the number of MIB and OR ≤ 1 breaks (first column) and the number of OR > 1 breaks (second column) in each of the samples (first row) compared to the same values in wild type (second row). The tables were formulated as below.

**Table:** 

A: the number of MIB and OR ≤ 1 breaks in the specific sample	B: the number of OR > 1 breaks in the specific sample
C: the number of MIB and OR ≤ 1 breaks in wild type	D: the number of OR > 1 breaks in wild type


**Table 1:** Fisher’s exact test *P*-values for determining any association between number of MIB and OR ≤ 1 breaks and repair defect.

Samples	Number of MIB and OR ≤ 1 breaks in a sample	Number of OR > 1 breaks in a sample	Number of MIB and OR ≤ 1 breaks in wild type	Number of OR > 1 breaks in wild type	*P*-values	OR	95% CI
Wild type treated with caffeine	879	557	750	1012	7.29E-26	2.13	1.84 – 2.46
Wild type day 50	2816	1728	750	1012	5.43E-44	2.20	1.96 – 2.46
*mec1Δ*	1939	114	750	1012	6.66E-294	22.93	18.53 – 28.59
*mre11Δ*	1855	234	750	1012	1.02E-214	10.69	9.04 – 12.67
*nej1Δ*	2742	2049	750	1012	6.30E-26	1.81	1.61 – 2.02
*rad51Δ*	1763	191	750	1012	1.32E-224	12.45	10.41 – 14.94
*tel1Δ*	3179	368	750	1012	5.44E-288	11.65	10.07 – 13.49
*yku70Δ*	2589	290	750	1012	7.27E-268	12.04	10.30 – 14.09
*yku80Δ*	1039	82	750	1012	1.95E-183	17.08	13.35 – 22.09


To determine whether MIB occurrence was associated with types of breaks, we performed Fisher’s exact tests from these contingency tables (Table 2). We calculated ten 2 × 2 contingency tables where A and B were the number of OR ≤ 1 breaks and OR > 1 breaks in reads not mapped from first base, respectively, C and D were the number of the number of OR ≤ 1 breaks and OR > 1 breaks in reads mapped from first base, respectively. Fisher’s exact test was then computed from these contingency tables.

**Table 2:** Fisher’s exact test *P*-values for determining any association between MIB occurrence and types of breaks.

Samples	Number of OR ≤ 1 breaks in reads not mapped from first base	Number of OR > 1 breaks in reads not mapped from first base	Number of OR ≤ 1 breaks in reads mapped from first base	Number of OR > 1 breaks in reads mapped from first base	*P*-values	OR	95% CI
Wild type	164	94	492	1012	1.82E-20	3.59	2.70 – 4.78
Wild type treated with caffeine	92	87	700	557	2.97E-01	0.84	0.61 – 1.17
Wild type day 50	518	424	1874	1728	1.07E-01	1.13	0.97 – 1.30
*mec1Δ*	846	949	144	114	9.38E-03	0.71	0.54 – 0.93
*mre11Δ*	669	932	254	234	7.45E-05	0.66	0.54 – 0.81
*nej1Δ*	548	387	1807	2049	1.26E-10	1.61	1.39 – 1.86
*rad51Δ*	587	933	243	191	1.43E-10	0.49	0.40 – 0.62
*tel1Δ*	1009	1757	413	368	2.83E-16	0.51	0.43 – 0.60
*yku70Δ*	1093	1145	351	290	9.34E-03	0.79	0.66 – 0.94
*yku80Δ*	429	555	55	82	4.63E-01	1.15	0.79 – 1.69


**FIGURE 3:**
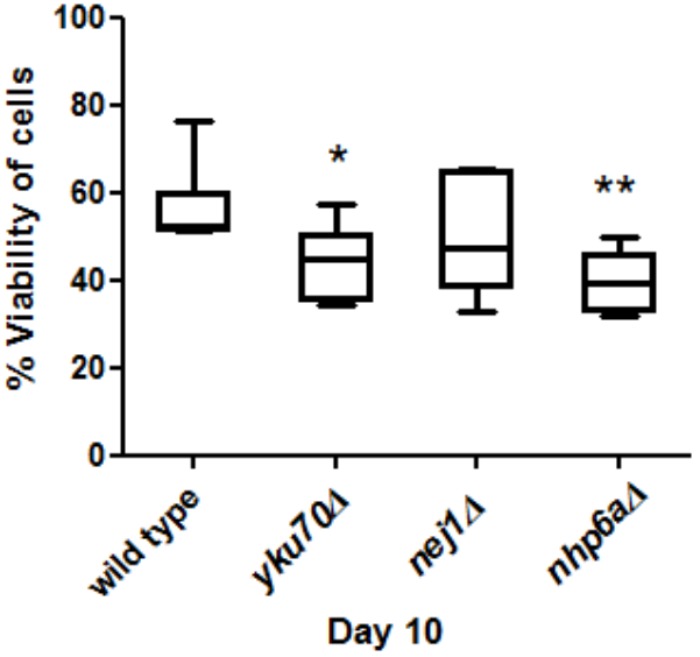
Comparisons of cell viability in *yku70Δ, nej1Δ, nhp6aΔ* and wild type at day 10. There was a significant decrease between *yku70Δ* and wild type (^∗^*P* < 0.05) and *nhp6aΔ* and wild type (^∗∗^*P* < 0.01).

Statistical analyses were performed using Student’s *t*-test for data with a normal distribution or a Mann–Whiney test for data that did not have a normal distribution, to determine (1) if % cell viability was significantly different between control and induced cells at each day (Figure 1A), (2) if the % differences were significantly different between day 10 vs. day 1, day 20 vs. day 1 and day 30 vs. day 1 (Figure 1B) and (3) if % viability was significantly different between wild type and each mutant (Figure 3).

## Results

### Chronological Aging Yeast May Have DNA Repair Defect

To show that chronological aging yeast has declined DNA repair capacity, we induced a break every 10 days in JKM179 by HO induction and observed whether those cells could repair the break by measuring the viability compared to control (cells with no induction). We found a significant % difference in viability between HO-induced JKM179 cells and the control cells (JKM179 cells with no induction) at days 20 and 30 (Figure 1A) and that % differences were significantly different between day 20 vs. day 1 and day 30 vs. day 1 (*p*-value < 0.05 and 0.001, respectively) (Figure 1B). Hence, we observed that aging cells are more sensitive to HO induction.

### Path-RIND-EDSBs (MIB and OR ≤ 1 Breaks) Increased in Cells With DNA Repair Defect

To explore if Path-RIND-EDSBs increased when DNA repair is defective, we looked at the proportion of the three break types, OR > 1 breaks, OR ≤ 1 breaks and MIB (see OR > 1 Breaks, OR ≤ 1 Breaks and MIB Calculation) in wild type, wild type treated with caffeine, wild type day 50, and seven repair defect yeast strains: *mec1Δ, mre11Δ, nej1Δ, rad51Δ, tel1Δ, yku70Δ*, and *yku80Δ*. The percentage of OR ≤ 1 breaks and MIB is significantly increased in all samples when compared to wild type (Figure 2 and Table 1). Wild type treated with caffeine and wild type day 50 had about the same increment of OR ≤ 1 breaks and MIB. All mutant except *nej1Δ* had similar increase of MIB which was much higher than both wild type treated with caffeine and wild type day 50. Therefore, we conclude that Path-RIND-EDSBs (OR ≤ 1 breaks and MIB) are increased in DNA repair defect cells.

### MIB Occurred More Frequently in OR ≤ 1 Breaks

We examined if there was an association between MIB and types of breaks (OR > 1 breaks and OR ≤ 1 breaks) (see Statistical Analyses). Results showed that the odds of MIB occurring at OR ≤ 1 breaks is 3.59 higher than no MIB in wild type (Table 2). On the contrary, in *mec1Δ, mre11Δ, rad51Δ, tel1Δ, yku70Δ*, the odds of MIB occurring at OR ≤ 1 breaks is lower than no MIB (OR = 0.71, 0.66, 0.49, 0.51, and 0.78, respectively, Table 2). In cells treated with caffeine, wild type day 50 and yku80Δ, MIB did not preferentially occur in either types of breaks. Therefore, we conclude that MIB tend to occur more frequently at Path-RIND-EDSBs in normal condition but occur at Phy-RIND-EDSBs in DNA repair defect.

### *yku70Δ* and *nhp6aΔ* Aged Faster Than Wild Type

Viability was used as a measure of aging to test if mutant strains aged faster than wild type. We grew *yku70Δ, nej1Δ, nhp6aΔ* and wild type cells for 10 days and then measured their viability at day 10. We found that the viability of *nhp6aΔ* and *yku70Δ* significantly decreased (*p* < 0.01 and *p* < 0.05, respectively) compared to wild type but not *nej1Δ* (Figure 3).

## Discussion

We found that chronological aging yeast has impaired DNA repair and found the accumulation of Path-RIND-EDSBs – as OR ≤ 1 breaks and MIB – increased in cells with DSB repair defects. Wild type treated with caffeine in this study had similar results to JKM179 treated with caffeine in the study of Thongsroy et al. (2018) in terms of the increment of proportion of OR ≤ 1 breaks and MIB. In mutant yeast with impaired DNA repair, Path-RIND-EDSBs, specifically MIB, increased even greater than cells treated with caffeine (DNA repair is defect by chemical). The results of wild type day 50 was similar to cells treated with caffeine. When we performed HO induction every 10 days, we found that % difference of cell viability between induced and non-induced cells increased significantly in aging cells compared to day 1 cells. These two results provide evidence that chronological aging cells may have DSB repair defect: firstly, RIND-EDSBs level in aging occur in the same direction as cells with repair inhibition; secondly, a reduction of viability of HO-induced cells compared to control increased as cells age.

DSB repair defects in chronological aging yeast may give rise to senescence phenotype by retention of Path-RIND-EDSBs. Pathologic EDSBs is a crisis state that requires immediate repair for cells. In response to DSB, players in damage response are activated, e.g., H2AX, p21, and p53 (Kulaberoglu et al., 2016; Ji et al., 2017). p53 triggers cell cycle arrest and promotes DSB repair. If damage cannot be repaired, apoptosis and/or senescence occurs (d’Adda di Fagagna, 2008), therefore, accumulation of DSBs can trigger senescence (Bielak-Zmijewska et al., 2018). We found that aging cells had Path-RIND-EDSBs retention due to DSB repair defect. Thus, we posit that DSB repair defect leads to senescence.

We postulate that a decline in repair in aging is due to the exhaustion of substrate of DNA repair. We previously found that Phy-RIND-EDSBs levels decreased in chronological aging yeast and associated with viability (Pearson correlation coefficient = 0.94) i.e., the lower the level of Phy-RIND-EDSBs, the lower the cells viability. This in turn increased the Path-RIND-EDSBs level. We observed an accumulation of Path-RIND-EDSBs in aging yeast cells (Thongsroy et al., 2018). Therefore, aging yeast cells have to repair Path-RIND-EDSBs more than younger cells. As cells age, more DNA repair machinery is required and DNA repair substrates are depleted resulting in DNA repair defect.

Aging cells have a decreased DNA repair efficiency; however, the repair mechanisms are not completely absent. While mutant strains had a markedly increase of MIB, aging cells had proportion of each breaks type in similar to caffeine treated cells. The number of MIB increased in aging cells but not at the same level as with mutant strains. This suggested that when cells are depleted of one of the repair proteins, they use an alternate repair pathway or their repair is compensated by some mechanism resulting in numerous MIB. Our results implied that MIB might be a mechanism that compensates repair defect (Table 2). We observed that MIB tended to occur at Path-RIND-EDSBs in wild type while the opposite was seen in repair defect cells: i.e., MIB tended to occur at Phy-RIND-EDSBs. This indicates that in normal condition, MIB might be an alternative pathway for Path-RIND-EDSBs, while Phy-RIND-EDSBs might have a specific mechanism to avoid MIB since they are retained in cells and need not be repaired immediately. However, when Phy-RIND-EDSBs cannot be repaired due to repair defect, MIB might be an alternative pathway as Phy-RIND-EDSBs must be repaired too. Another possibility is MIB might be an end modification process that occurred after DSB repair defect. This end modification process might leads to cell death i.e., its role might be to tag the damage sites to be recognized by certain apoptosis pathway, similar to ubiquitin tagging in protein degradation pathway (Pickart, 2004). Another possibility is the end modification process might prevent cell death i.e., its role might be to disguise Phy-RIND-EDSBs from apoptosis pathway just as telomere ends that were protected from damage response pathway (de Lange, 2009).

Nej1 was either not involved in repairing RIND-EDSBs or it might have other proteins with a redundancy role. Our results showed that its Path-RIND-EDSBs level increased slightly: *nej1Δ* results were very different from other mutant strains and this was in accord with (Thongsroy et al., 2013). They observed no change in RIND-EDSBs level in nej1Δ but the level was significantly increased in *mec1Δ, tel1Δ, mre11Δ, rad51Δ, yku70Δ* and *yku80Δ*. Therefore, DSB repair complex of Path-RIND-EDSBs might be different from other pathologic DSBs. Results from Yang et al. (2015) perhaps support the possibility that Nej1 is compensatory. Nej1 together with Dnl4/Lif1 carried out DNA end joining (Pannunzio et al., 2014). Both Nej1 and Dnl4/Lif1 additively but independently recruit end processing factors, Pol4 and Rad27. They found that no recruitment of Pol4 and Rad27 was observed in *lif1Δnej1Δ*. However, loss of either Nej1 or Dnl4/Lif1 reduced the rate of recruitment. They also showed that flap cleavage and ligation product were detected in reactions that lack Nej1. However, when Nej1 was added, the product was enhanced, indicating a stimulatory effect of Nej1.

Our finding that *yku70Δ* and *nhp6aΔ* aged faster than wild type is in agreement with some other studies. Ju et al. (2006) showed that expression of Ku70 in the longevity group was higher than in control group suggesting that people who possess higher level of Ku70 expression have a longer life span. Li et al. (2007) found that Ku70, Ku80 mutant and double-mutant mice exhibit early aging with very low cancer levels. Giavara et al. (2005) found that *nhp6a/b* mutant strain had a significantly shorter lifespan than *NHP6A/B*. The results of *nej1Δ* that a significant decrease of viability at day 10 was not observed is perhaps consistent with our other finding that pathologic RIND-EDSBs level increased slightly in *nej1Δ*.

## Conclusion

Our data showed an accumulation of Path-RIND-EDSBs in aging cells and cells with repair defects either chemically defected (caffeine) or genetically defected (mutant strains). In addition, a more pronounced increment of % difference of cell viability in aging cells was observed when cells were induced with HO. These support the conclusion that chronological aging yeast might have impaired DNA repair. MIB is employed to process Path-RIND-EDSBs in normal condition; however, it is used to handle Phy-RIND-EDSBs if repair is defective. Since DSB repair defect might lead to senescence phenotype, restoring repair defects is an important aim to slow down deterioration of cells from chronological aging.

## Data Availability Statement

The datasets analyzed for this study can be found in the Sequence Read Archive under the accession numbers SRP095646.

## Author Contributions

MoP performed all of the bioinformatics analyses and wrote the manuscript. MaP prepared the samples for sequencing and helped to draft the manuscript. AM conceived and designed the study, analyzed the data, and edited the manuscript. All the authors have read and approved the final manuscript.

## Conflict of Interest Statement

The authors declare that the research was conducted in the absence of any commercial or financial relationships that could be construed as a potential conflict of interest.
